# Dynamics of central and peripheral immunomodulation in a murine glioma model

**DOI:** 10.1186/1471-2172-10-11

**Published:** 2009-02-18

**Authors:** Benjamin C Kennedy, Lisa M Maier, Randy D'Amico, Christopher E Mandigo, Elizabeth J Fontana, Allen Waziri, Marcela C Assanah, Peter Canoll, Richard CE Anderson, David E Anderson, Jeffrey N Bruce

**Affiliations:** 1 Department of Neurosurgery, Gabriele Bartoli Brain Tumor Research Laboratory Columbia University, New York, NY, USA; 2 Center for Neurologic Diseases, Department of Neurology, Brigham and Women's Hospital and Harvard Medical School, Boston, MA, USA; 3 Department of Neurosurgery, University of Colorado Health Sciences Center, Denver, CO, USA; 4 Department of Pathology, Columbia University, New York, NY, USA

## Abstract

**Background:**

Immunosuppression by gliomas contributes to tumor progression and treatment resistance. It is not known when immunosuppression occurs during tumor development but it likely involves cross-talk among tumor cells, tumor-associated macrophages and microglia (TAMs), and peripheral as well as tumor-infiltrating lymphocytes (TILs).

**Results:**

We have performed a kinetic study of this immunomodulation, assessing the dynamics of immune infiltration and function, within the central nervous system (CNS) and peripherally. PDGF-driven murine glioma cells were injected into the white matter of 13 mice. Four mice were sacrificed 13 days post-injection (dpi), four mice at 26 dpi, and five mice at 40 dpi. Using multiparameter flow cytometry, splenic T cells were assessed for FoxP3 expression to identify regulatory T cells (Tregs) and production of IFN-γ and IL-10 after stimulation with PMA/ionomycin; within the CNS, CD4^+ ^TILs were quantified, and TAMs were quantified and assessed for TNF-α and IL-10 production after stimulation with LPS. Peripheral changes associated with tumor development were noted prior to effects within the CNS. The percentage of FoxP3^+ ^regulatory T cells (Tregs) increased by day 26, with elevated frequencies throughout the duration of the study. This early increase in Tregs was paralleled by an increase in IL-10 production from Tregs. At the final time points examined (tumor morbidity or 40 dpi), there was an increase in the frequency of TAMs with decreased capacity to secrete TNF-α. An increase in TIL frequency was also observed at these final time points.

**Conclusion:**

These data provide insight into the kinetics of the immunosuppressive state associated with tumor growth in a murine model of human gliomas. Functional impairment of TAMs occurs relatively late in the course of GBM tumor growth, potentially providing a window of opportunity for therapeutic strategies directed towards preventing their functional impairment.

## Background

Despite decades of research and clinical experience with surgical, radiotherapeutic, chemotherapeutic, and other therapy modalities, prognosis for malignant glioma has remained dismal, with median survival for WHO Grade IV tumors still being approximately one year despite aggressive treatment [1,2]. Immunotherapy represents a theoretically promising treatment [3-6], though early experiences with glioma immunotherapy have been disappointing [7,8]. In the past, these disappointments would have been attributed to the "immune privilege" of the brain [9], citing the relative impermeability of the blood-brain barrier to immune cells, lack of CNS lymphatics, and the relative immunoincompetence of microglia, the resident CNS macrophage. However, more recently, evidence of immunity to exogenous as well as endogenous antigens in the CNS [10] suggests that the interaction between the CNS and immune system is much more complex [11,12]. Immunosuppression by gliomas may contribute to tumor progression and treatment resistance. It is not known when immunosuppression occurs during tumor development but it likely involves cross-talk among tumor cells, tumor-associated macrophages and microglia (TAMs), and peripheral as well as tumor-infiltrating lymphocytes (TILs) [13,14].

In humans, we and others [15,16] have observed that up to a third of the GBM tumor mass can consist of TAMs. However, these GBM-infiltrating TAMs are reported to be functionally impaired [16-19]. As these cells represent the large immune infiltrate in human gliomas, understanding the mechanisms by which TAMs are rendered non-functional represents an important step in developing effective immunotherapy for this disease.

We and others have shown in humans and animal models that the lymphocyte population from within gliomas is larger than in unaffected brain, and exhibits functional impairment as well [20-22] (CEM, unpublished observations). Glioma-associated immunosuppression has also been observed in peripheral immune cell populations, notably Tregs, a T cell population that plays a role in the maintenance of tolerance to self antigen [23]. Evidence suggests that Tregs may play an important role in the maintenance of self-tolerance in neoplastic disease, including GBM [21,24-27].

The dynamics by which this immunomodulation occurs has not been systematically studied throughout the course of tumor development. Humans cannot be studied until clinical diagnosis, a relatively late timepoint in the disease. There is also a paucity of animal research describing the kinetics of such effects [19]. Accordingly, using a PDGF-driven syngeneic murine glioma model, we have performed a kinetic study of this immunomodulation, assessing the dynamics of immune infiltration and function, both within the tumor and peripherally.

## Results

**Figure 1:**
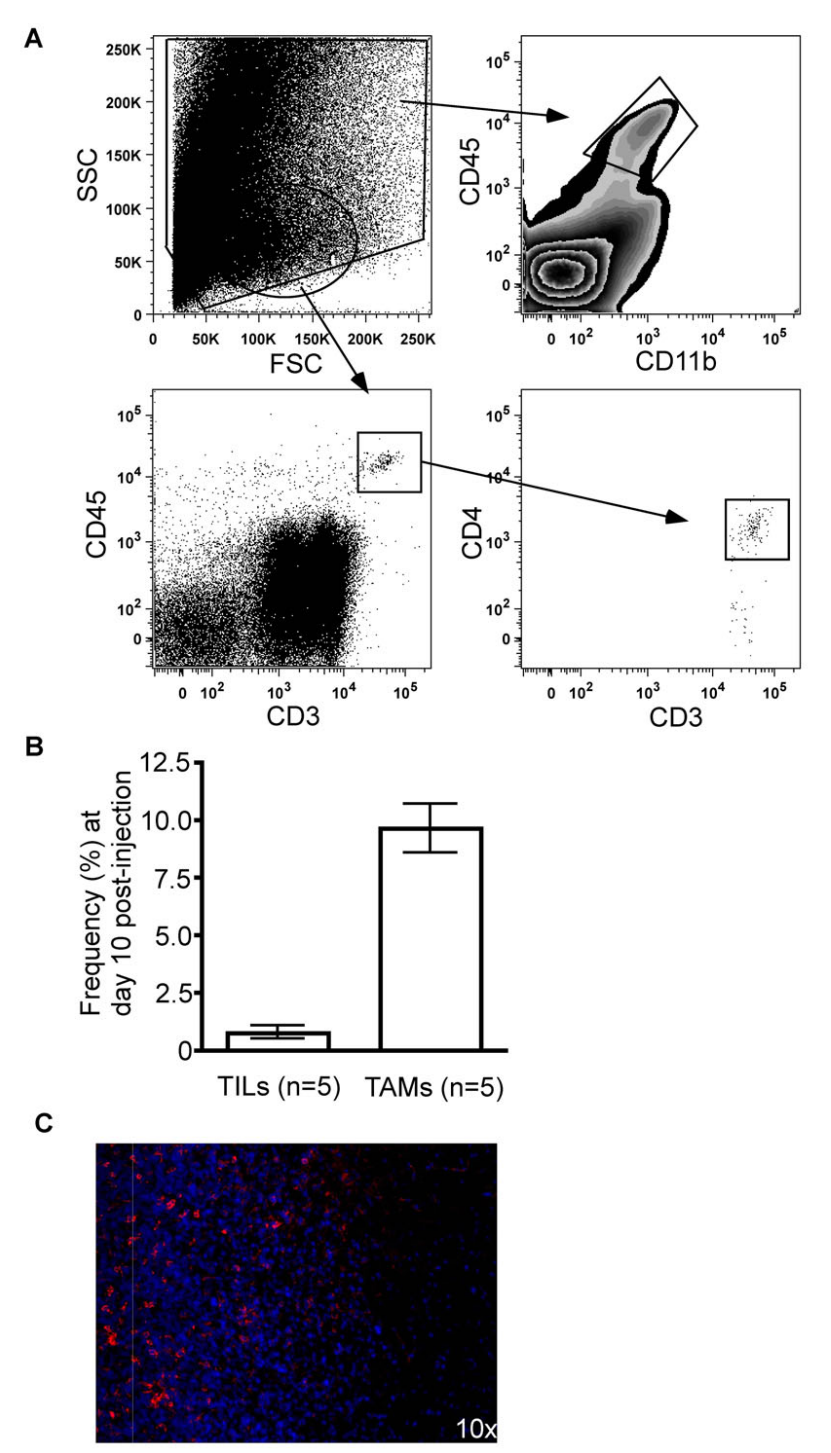
**Detection of tumor-infiltrating lymphocytes (TILs) and tumor-associated macrophages and microglia (TAMs) in *ex vivo *tumor specimens**. (A) Flow cytometryic analysis and gating strategy used to detect TILs (CD3^+^CD45^+ ^cells) and TAMs (CD11b^+^CD45^+ ^cells) in brain. The initial gate excludes data points with extreme side and forward scatter properties. (B) The frequency of TILs and TAMs as obtained by flow cytometry was increased in *ex vivo *tumor specimen. Note that the TAMs shown here were detected by CD45. (C) Immunofluorescent staining of *ex vivo *tumor specimen revealed a large number of TAMs in the tumor specimen as compared to neighboring white matter. Rhodamine (red) staining for CD11b indicating TAMs, Hoescht (blue) staining for nuclei. The increased cellularity on the left side of the image is grossly visible tumor tissue, and the right side of the image is white matter without gross tumor involvement.

In an initial series of experiments, we aimed to determine the relative frequencies of tumor-infiltrating lymphocytes (TILs) versus tumor-associated macrophages/microglia (TAMs). In this preliminary study, TAMs were defined as cells isolated from 10 dpi *ex vivo *tumor specimens expressing CD45 but not expressing CD3; in our more extensive follow up study, however, we defined TAMs as cells expressing CD45 and CD11b, a marker for cells of myeloid lineage that includes monocytes, macrophages, and microglia (cells expressing these markers comprise potential antigen-presenting cells (APCs) within the tumor bed). TILs were defined as CD3^+^CD45^+^, and may include a variety of T cell subsets, including both CD4^+ ^T helper cells and CD8^+ ^cytotoxic T lymphocytes (CTLs). After excluding cells with extreme side and forward scatter properties, the frequencies of these two cell populations were quantified (Figure 1.A). Noteworthy was the much higher frequency of TAMs relative to TILs in all animals examined (Figure 1.B). Not surprisingly, greater numbers of TILs were apparent when there were also greater numbers of TAMs, suggesting a correlation between infiltration of both adaptive (TIL) and innate (TAM) cell populations.

While use of multiparameter flow cytometric analysis of *ex vivo *tissue specimens gives more quantitative measurements of cell populations than traditional immunohistochemical techniques, the anatomical location of the cell populations of interest are lost. Accordingly, we performed immunofluorescent staining of tissue that included the tumor/parenchyma border, in order to determine better where TAMs were located in tumor-bearing mice. Immunofluorescent staining of CD11b in animals with late stage disease shows a robust population of TAMs along the tumor border and within the highly cellular tumor (Figure 1.C).

While our initial experiments demonstrated that TAMs outnumbered TILs early after tumor implantation, it remained unclear whether this was true throughout the course of tumor growth. Furthermore, we were interested to test if these cells maintained their functional activity, and what immunological changes were occurring systemically. This last question was of interest given that increases of Tregs have been observed in many tumors, including GBMs [22,25-29](CEM, unpublished observations), and there is evidence that Tregs may function in part by suppressing APC activity. We thus performed a more comprehensive study that investigated immunological changes both systemically and within the tumor microenvironment, which included an evaluation of the function of TAMs isolated from *ex vivo *tumor specimens. Mice were examined after tumor implantation at early (day 13), intermediate (day 26), and late (day 40 or signs of tumor morbidity) stages of disease.

**Figure 2:**
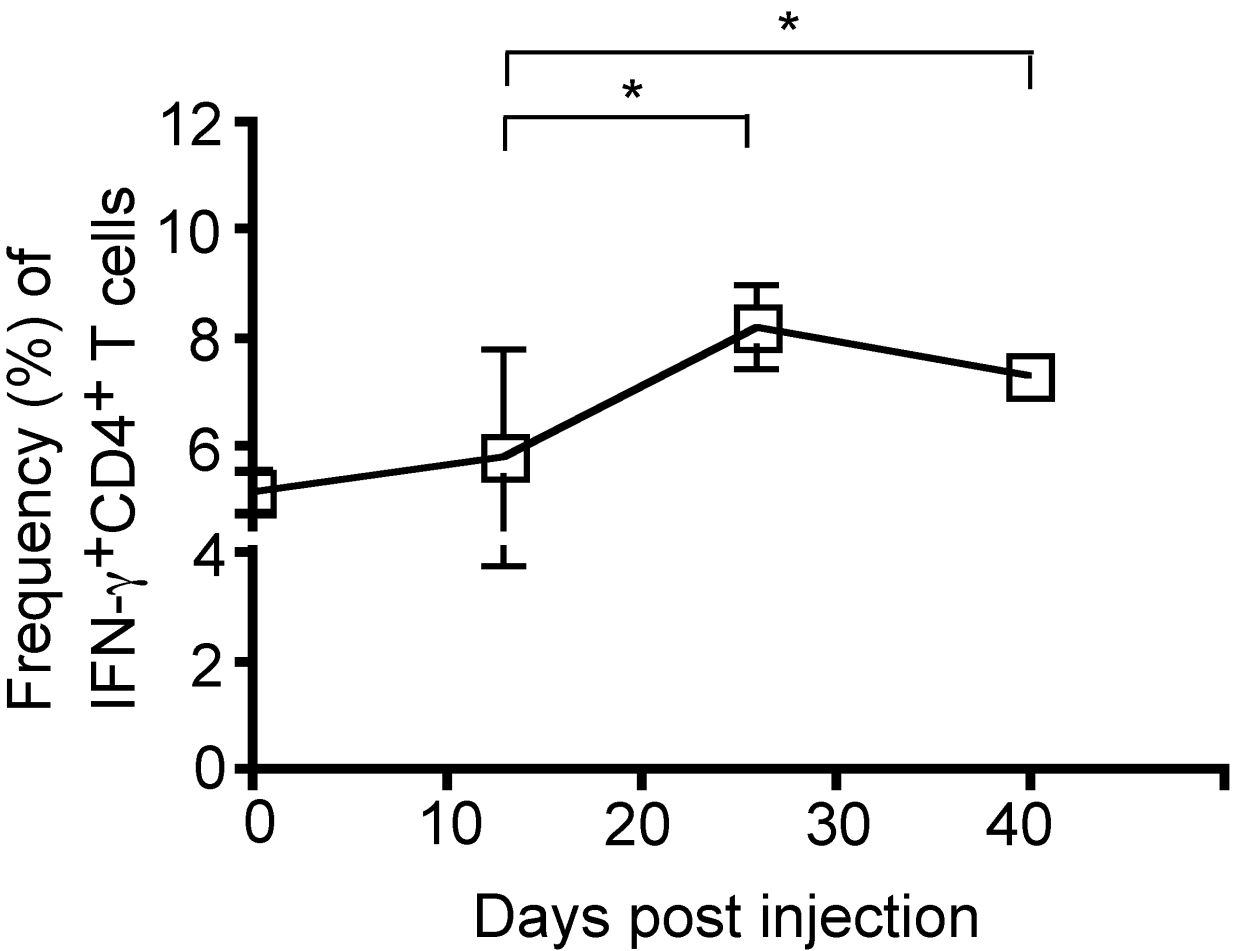
**Peripheral immunomodulation over the course of tumor growth**. Splenocytes were stained for extracellular CD4 and intracellular IFN-g expression. The proportion of CD4^+ ^T cells expressing IFN-γ changes over the course of tumor development. Between the early and intermediate timepoint, the proportion of IFN-γ-producing splenic CD4^+ ^T cells increased (5.1% vs. 8.2%, *P *= 0.015), and remained elevated (5.1% vs. 7.7%, *P *= 0.011). 13 mice were analyzed at 13 dpi (n = 4), 26 dpi (n = 4), and at 29 dpi upon exhibiting clinical tumor morbidity (n = 1) or at 40 dpi (n = 4). Differences between the means at each time point were tested using two-sided t tests with unequal variances. *, *P *< 0.05; **, *P *< 0.01, ***, *P *< 0.001.

**Figure 3:**
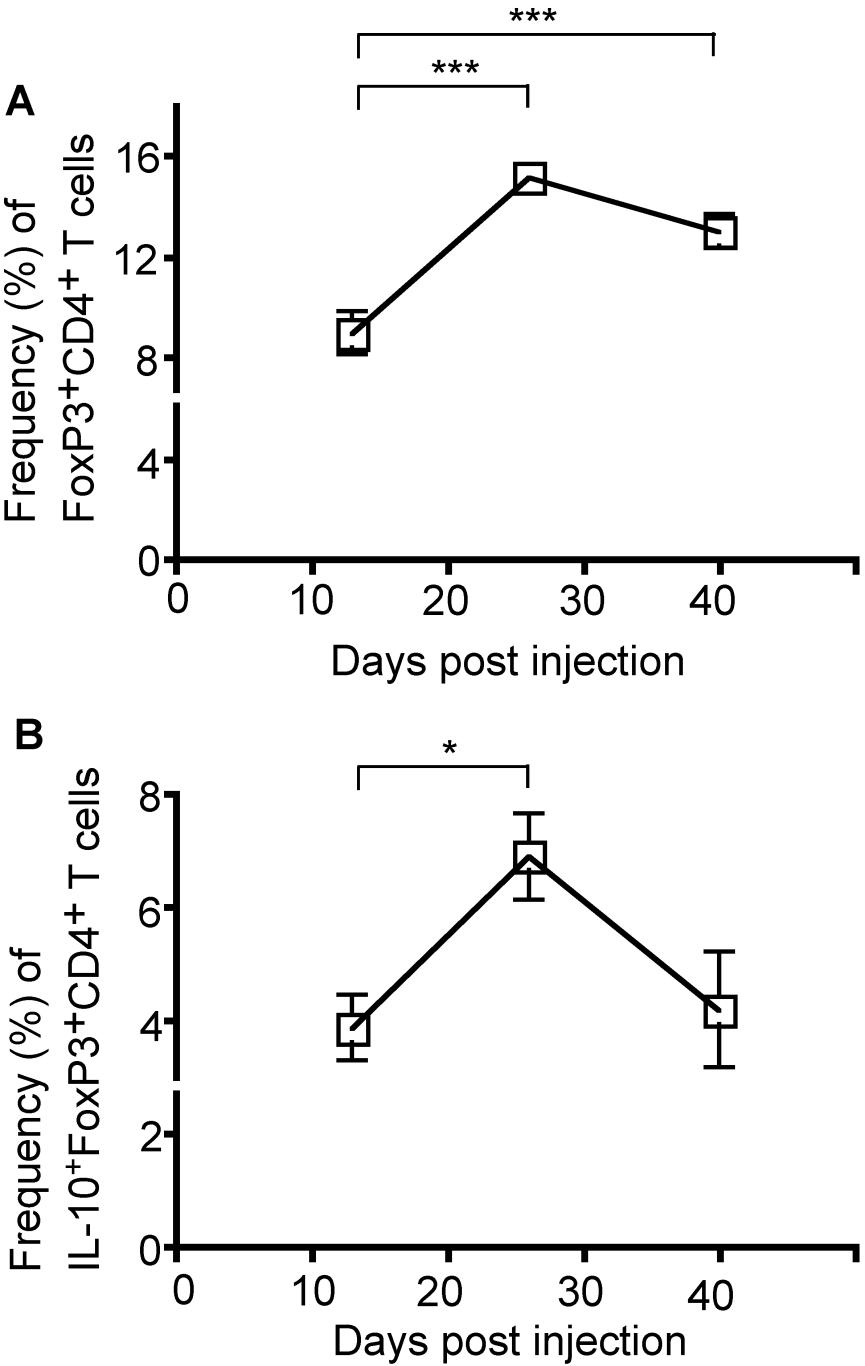
**Peripheral changes in frequency and function of CD4^+^FoxP3^+ ^Treg cells**. Splenocytes were stained for extracellular CD4 and intracellular FoxP3 and IL-10 expression. (A) An increase in Treg cell frequency among CD4+ T cells was observed between the early and intermediate time points (9.0% vs. 15.2%, *P *= 0.0007) and this frequency remained elevated at the late time point. (B) While the proportion of Tregs expressing IL-10 increased between the early and intermediate time points (3.9% vs. 6.9%, *P *= 0.02), the proportion of IL-10-producing Tregs at the late time point returned to levels measured at the early time point (4.2%). 13 mice were analyzed at 13 dpi (n = 4), 26 dpi (n = 4), and at 29 dpi upon exhibiting clinical tumor morbidity (n = 1) or at 40 dpi (n = 4). Differences between the means at each time point were tested using a Mann-Whitney test. *, *P *< 0.05; **, *P *< 0.01, ***, *P *< 0.001.

Within the periphery, we assessed the IFN-γ production in T cells, as this is the major immunostimulatory cytokine in this cell population. There was a modest increase in the proportion of IFN-γ-producing CD4^+ ^T cells (5.1% vs. 8.2%, *P *= 0.015) between the early and intermediate time points examined, with these frequencies elevated through the course of disease (5.1% vs. 7.7%, *P *= 0.011) (Figure 2). In contrast, more profound changes were observed in the frequency and function of Tregs (CD4^+^FoxP3^+ ^T cells) in the periphery (Figure 3). The proportion of Tregs among CD4^+ ^T cells increased substantially between the early and intermediate time points (9.0% vs. 15.2%, *P *= 0.0007), and remained elevated, though with a slight decrease from the peak frequency, throughout the course of disease (Figure 3A). Noteworthy was that the proportion of Tregs expressing IL-10, the major immunoinhibitory cytokine in this cell population, increased between the early and intermediate time points (3.9% vs. 6.9%, *P *= 0.020), with a decrease in this frequency by the final time point (6.9% vs. 4.2%, *P *= 0.080) (Figure 3B).

**Figure 4:**
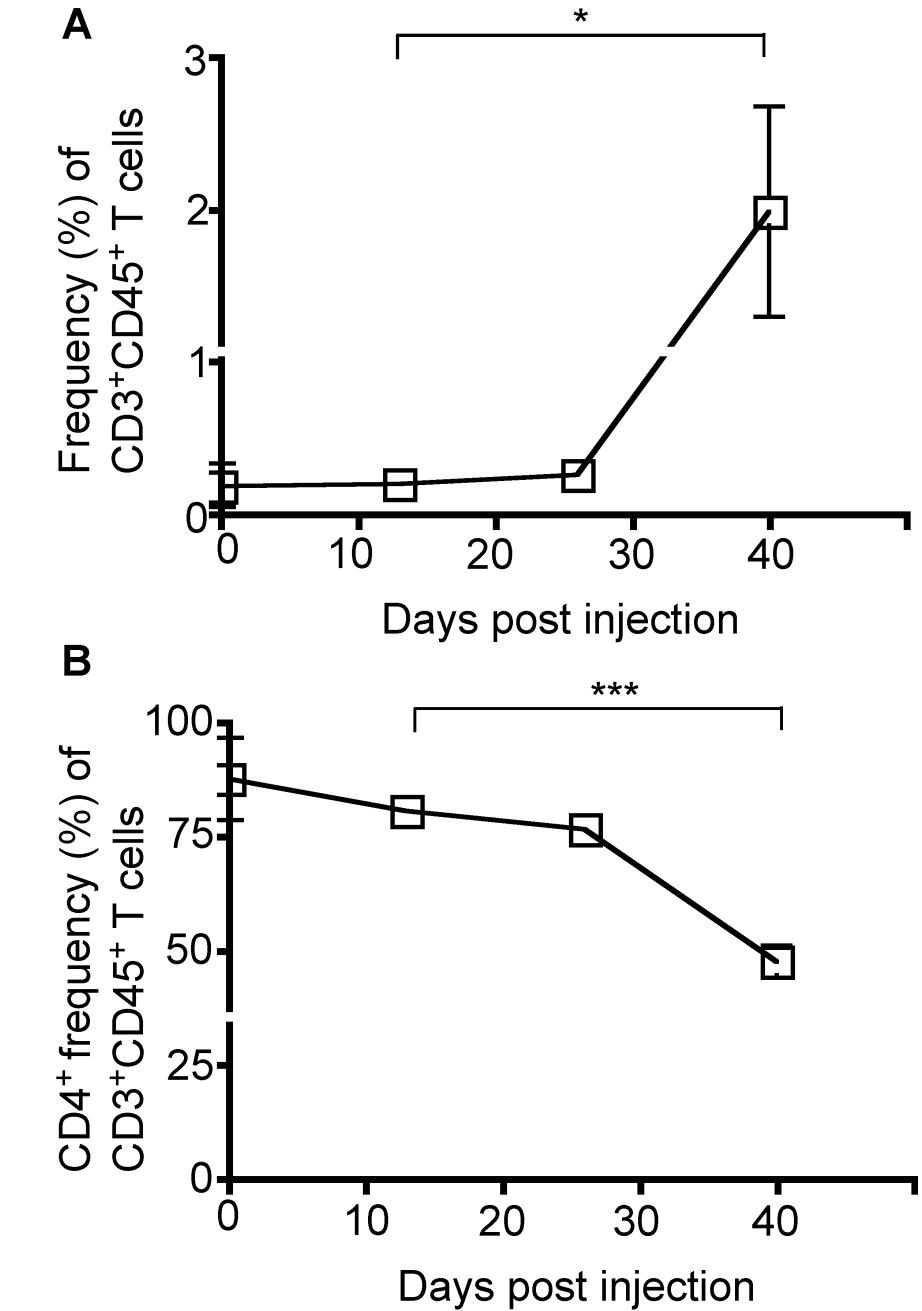
**Frequency of tumor-infiltrating lymphocytes (TILs) in *ex vivo *tumor specimens over the course of tumor development**. Specimens were obtained prior to and at three time-points following tumor induction and stained for CD3, CD4, and CD45 expression. (A) An increase in the frequency of TILs as characterized by CD3 and CD45 expression was observed at the late time-point (0.3% vs. 2.0%, *P *= 0.047). (B) The percentage of CD4^+ ^T cells among TILs was reduced at the late time point (76.5% vs. 47.5%, *P *= 0.0003). 13 mice were analyzed at 13 dpi (n = 4), 26 dpi (n = 4), and at 29 dpi upon exhibiting clinical tumor morbidity (n = 1) or at 40 dpi (n = 4). Differences between the means at each time point were tested using two-sided t tests with unequal variances. *, *P *< 0.05; **, *P *< 0.01, ***, *P *< 0.001.

TILs were defined as cells present within *ex vivo *tumor specimens that expressed both CD3 and CD45 (Figures 1 and 4). Between the early and intermediate time points, there were no observed changes in TIL frequency (0.2% vs. 0.3%) (Figure 4A). However, later in the course of disease, when comparing the intermediate and late time points, an increase in TIL frequency was observed (0.3% vs. 2.0%, *P *= 0.047) (Figure 4A). Among these CD3^+^CD45^+ ^TILs, we observed a decrease between the early/intermediate and the late time point in the proportion of TILs that were CD4^+ ^(76.5% vs. 47.5%, *P *= 0.0003), suggesting that alternate T cell subsets may be infiltrating the tumor microenvironment, such as CD8^+ ^CTLs or NK T cells (Figure 4B).

**Figure 5:**
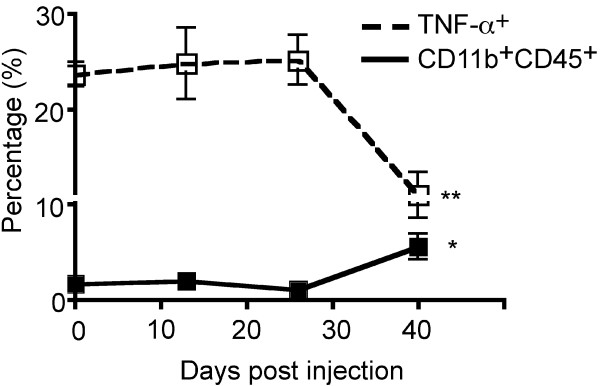
**Frequency and functional changes in tumor-associated macrophages and microglia (TAMs) in *ex vivo *tumor specimens over the course of tumor development**. While the percentage of TAMs (CD11b^+^CD45^+ ^cells) was increased by the final time-point (1.1% vs. 5.6%, *P *= 0.017), their functional capacity was impaired as measured by TNF-a expression (25.2% vs. 10.9%, *P *= 0.007). 13 mice were analyzed at 13 dpi (n = 4), 26 dpi (n = 4), and at 29 dpi upon exhibiting clinical tumor morbidity (n = 1) or at 40 dpi (n = 4). Differences between the means at each time point were tested using two-sided t tests with unequal variances. *, *P *< 0.05; **, *P *< 0.01, ***, *P *< 0.001.

We again quantified the frequencies of TAMs, this time over the course of tumor growth and with the more specific APC-marker combination of CD45 and CD11b. Moreover, these cells were assessed for secretion of TNF-α and IL-10, the major immunostimulatory and immunoinhibitory cytokines from this cell population, respectively. Between the early and intermediate time points, very little change in TAM frequency was observed (2.0% vs. 1.1%). Between the intermediate and final time points, however, there was a substantial increase in frequency of TAMs (1.1% vs. 5.6%, *P *= 0.017) (Figure 5). Of greatest interest, there was no observed change in the proportion of TAMs secreting TNF-α *ex vivo *between the early and intermediate time points (24.7% vs. 25.2%), but at the late stages of tumor growth, a 2.5-fold reduction in the proportion of these TAMs expressing TNF-α was observed (25.2% vs. 10.9%, *P *= 0.007) (Figure 5). No changes in IL-10 expression were detected (data not shown).

## Discussion

In this study we report on the dynamics of central and peripheral immunomodulation in a murine glioma model. The most notable change within the tumor microenvironment was an increase in TAM frequency over the course of tumor growth, which correlated with functional impairment, demonstrated by a decreased proportion of TNF-α-secreting cells after mitogenic stimulation of *ex vivo *TAMs. TIL frequency also increased during tumor growth, with a decline in the relative proportion of CD4^+ ^T cells among the TIL population. In the periphery, consistent with other tumor models that include glioma models [22,26,28,29], the proportion of Tregs in the spleen increased substantially relatively early in tumor development, which was accompanied by increased frequencies of IL-10-expressing Tregs. There was a modest increase in IFN-γ-secreting CD4^+ ^T cells throughout the course of tumor growth.

Prior studies in humans and animal models have shown the frequency of TAMs to be higher than that of APCs in non-tumor-bearing brain [15] (DEA, unpublished observations). Our findings are consistent with these observations, and may represent local microglial proliferation, microglial migration into the tumor, and infiltration by peripheral monocytes with subsequent differentiation to a macrophage or microglial phenotype. Previous studies have demonstrated that TAMs have an immunosuppressive phenotype, unable to promote T cell activation [16]. Our study has extended these observations by demonstrating that a tumoricidal function of TAMs, namely secretion of TNF-α, is lost at a relatively late point in tumor growth.

Tregs have been implicated in the complex immunomodulatory phenomena of gliomas [22,26,28,29], CEM unpublished observations). Prior studies in humans have shown an increased fraction of Tregs centrally and peripherally [21,24,25], but it is unclear if this is an early event, with a potential for assisting in tumor progression, or a late event, suggesting that the Tregs may be epiphenomenological. Our study suggests that the peripheral Treg population is increased relatively early in tumor development, raising important questions about the role of these cells in tumor progression. Previous studies have shown TILs to consist of a higher proportion of Tregs compared to CNS lymphocytes not associated with gliomas [27]. Although this was not assessed in the present study, we did observe that the relative proportion of CD4^+ ^T cells among TILs decreased over time. Although the frequency of CD8^+ ^T cells was not addressed in these samples, it is likely that the overwhelming majority of these CD4^- ^TILs are CD8^+ ^T cells. It will be important in future studies to determine if these are indeed CTLs, or an alternate population of immunosuppressive T cells [30].

The increase of Treg frequency observed in this study occurs relatively early compared to the observed CNS effects. In fact, between the intermediate and final time points examined in the course of tumor growth, when all observed CNS changes occur, the peripheral Treg proportion actually decreases, though still elevated from baseline. A similar effect is seen in the proportion of Tregs expressing IL-10. These data suggest that the peripheral immunosuppressive effects may occur prior to the local effects present in the tumor microenvironment. Indeed, it is tempting to speculate that tumor infiltrating Tregs may contribute to the loss of TAM function later in the course of disease.

## Conclusion

In conclusion, this study represents to our knowledge the first detailed kinetic analysis of the frequency and function of TAMs present in gliomas. Our discovery that the function of TAMs is not affected until relatively late in tumor growth suggests that a therapeutic window may exist in which to preserve the function of these cells, and potentially have a positive clinical effect on glioma outcome.

## Methods

### Tumor Model

All animal care and experiments involved with this work follow internationally recognized guidelines and are overseen by the Columbia University Institutional Animal Care and Use Committee (Columbia University IACUC Protocol # AC-AAAA6721).

Several studies have shown that PDGF-expressing retrovirus will induce the formation of glial tumors in mice [31,32]. Our lab has generated a murine glioma model, similar to our rat model [33], by injecting PDGF-expressing retrovirus into the white matter of adult BALB/c mice (CEM, unpublished). Our reasons for use of this model are several. Use of a syngeneic tumor model such as ours is essential to appropriately characterize the interactions of the tumor with the immune system, as syngeneity confers MHC expression recognizable by the animals' adaptive immune systems, as well as avoids induction of an immune response to exogenous foreign antigen. Histologically, these tumors closely resemble GBM, demonstrating palisading necrosis, vascular proliferation, and migration and infiltration by the tumor cells. Considering the high frequency of immune cells at the borders of these tumors, these infiltrative characteristics make this a particularly strong model for gaining insight into the interactions between GBM and the immune system. We have shown that these tumor cells can be isolated and injected into naïve mice for several generations, producing consistent tumor formation.

Briefly, a total of 1 × 10^5 ^tumor cells were injected into the right frontal white matter of 13 adult BALB/c mice. All mice were female, between 7 and 8 weeks old, and purchased from Jackson Laboratory (Bar Harbor, ME). In the first series of experiments, 4 mice were injected with tumor cells and then analyzed at 10 dpi. In a subsequent, more comprehensive series of experiments, 13 mice were analyzed at 13 dpi (n = 4), 26 dpi (n = 4), and at 29 dpi upon exhibiting clinical tumor morbidity (n = 1) or at 40 dpi (n = 4); the mouse that became symptomatic at 29 dpi was included with the animals studied at 40 dpi. Mice were anesthetized with ketamine and xylazine, sacrificed, and spleens and brains were harvested.

### Splenocyte Preparation

Fresh spleens were dissociated into single cell suspensions through a 70 μm filter in culture medium (MEM alpha medium with 2ME, Penicillin/Streptomycin/Amphotericin, and 10% FBS). Culture medium and supplies were purchased from Invitrogen (Carlsbad, CA). Cells were stimulated with 5 nM PMA, 1 mM Ionomycin, and 0.1% monensin solution (eBioscience, San Diego, CA) and incubated at 37°C for 9 hours.

### Tumor Preparation

Tumors were dissected and mechanically as well as enzymatically dissociated with trypsin, papain, and DNAse. Samples were filtered through 70 μm mesh to create single cell suspensions. These suspensions were purified using a sucrose gradient at 2000 rpm for 20 min to remove neurons and blood, and resuspended. Cells were stimulated with 1 μM LPS and 0.1% monensin and incubated at 37°C for 7.5 hrs.

### Flow Cytometry

Splenocytes and tumor cell preparations were washed with PBS/2% FBS and 0.01% monensin, spun again, and resuspended in 100 μl of this medium. Cell surface staining was performed at room temperature, in the dark, for 20 min. Splenocytes were stained using FITC-conjugated anti-mouse CD8, PerCP-conjugated anti-mouse CD3, and Pacific Blue-conjugated anti-mouse CD4 monoclonal antibodies. Tumor cell preparations were stained using PE-conjugated anti-mouse CD11b, PerCP-conjuaged anti-mouse CD45, APC-conjugated anti-mouse CD3, Pacific Blue-conjugated anti-mouse CD4, and Pacific Blue-conjugated anti-mouse CD3 monoclonal antibodies. Cells were washed once in PBS/2% FBS and 0.1% monensin, then resuspended in 200 μL of Fix/Perm solution (BD Bioscience, San Jose, CA) at 4°C for 30 min. They were then washed twice in permeabilization buffer (BD Bioscience, saponin, FBS), and stained for cytokines and FoxP3 at 4°C for 30 min. For intracellular stainining, splenocytes were stained using PE-FoxP3, APC-IFN-γ, and APC-IL-10 antibodies. Tumor cell preparations were stained using APC-conjugated anti-mouse TNF-a and APC-conjugated anti-mouse IL-10 antibodies. The cell preparations were then washed twice in Perm buffer (BD Bioscience) and resuspended in PBS/2% FBS. All antibodies for intracellular and cell surface staining were purchased from eBioscience (San Diego, CA), except for PerCP-conjugated anti-mouse CD45, which was purchased from BD Bioscience (San Jose, CA).

Multiparameter flow cytometry data were collected on an LSR II (BD Biosciences) and analyzed using FlowJo version 8.7.1 (Tree Star, Ashland, OR). As lymphocytes are a large proportion of the splenocyte preparation, there is a reliable and recognizable cluster with the expected forward and side scatter properties, around which the initial gates were created. However, due to the relatively small population of TILs within the tumor/CNS preparation, serial dilutions of splenocytes with tumor preparations were made to derive a lymphocyte gate in tumor preparations. This gate was then applied to the pure tumor preparations for all TIL analyses.

### Immunofluorescence

In separate BALB/c mice injected with the same tumor cells in the same fashion outlined above, tumors were grown until tumor morbidity was apparent. Mice were then anesthetized with ketamine and xylazine, sacrificed, perfused with PBS, then PBS with 4% paraformaldehyde. Brains were harvested and kept in 4% paraformaldehyde for 24 hours, then stored in increasing concentrations of glucose in PBS up to 30%. The brains were then frozen, sliced, and mounted on glass slides. For indirect immunofluorescence labeling, sections were pre-incubated with buffered blocking solution (PBS containing 10% horse serum, 0.5% triton and NaN_3 _in dilution of 1:500) for 1 hr at room temperature. Sections were subsequently incubated with rat anti-mouse CD11b primary antibody (eBioscience, San Diego, CA) diluted 1:100 in buffered blocking solution at room temperature overnight. Subsequently, sections were thoroughly washed (5 × 3 mins) in PBS and incubated in solutions of rhodamine-conjugated goat anti-rat IgG secondary antibody (Jackson Immunological, West Grove, PA) in buffered blocking solution for 1 hr at room temperature. After incubation, sections were thoroughly washed again in PBS (5 × 3 mins) and incubated with Hoescht stain for five minutes for nuclear visualization. Sections were washed in PBS again and rinsed with distilled water.

### Statistical Analyses

Where appropriate, means of proportions were compared using two-tailed Student's t-tests with unequal variances. For analyses of small proportions (< 0.05), logarithmically transformed data were analyzed using one-way ANOVA, and parameters with statistically significant ANOVA results were analyzed further using two-tailed Student's t-tests. P-values less than 0.05 were considered significant for all analyses.

## Abbreviations

TILs: Tumor-Infiltrating Lymphocyte; TAMs: Tumor-Associated Macrophages/Microglia; APCs: Antigen-Presenting Cells; GBM: Glioblastoma Multiforme.

## Authors' contributions

BCK provided flow cytometric data, performed immunoassays, developed study design, and wrote the manuscript. LMM performed analyses of flow cytometric data and wrote the manuscript. RD performed immunohistochemical staining. CEM provided the mouse model. EJF helped with flow cytometry. AW and MCA contributed to study coordination. PC helped develop overall study design. RCEA, DEA, and JNB conceived the study, participated in study design and coordination, and wrote the manuscript.
